# Diversity of Plasmids and Genes Encoding Resistance to Extended Spectrum Cephalosporins in Commensal *Escherichia coli* From Dutch Livestock in 2007–2017

**DOI:** 10.3389/fmicb.2019.00076

**Published:** 2019-02-04

**Authors:** Daniela Ceccarelli, Arie Kant, Alieda van Essen-Zandbergen, Cindy Dierikx, Joost Hordijk, Ben Wit, Dik J. Mevius, Kees T. Veldman

**Affiliations:** ^1^Department of Bacteriology and Epidemiology, Wageningen Bioveterinary Research, Lelystad, Netherlands; ^2^Netherlands Food and Consumer Product Safety Authority (NVWA), Utrecht, Netherlands; ^3^Faculty of Veterinary Medicine, Utrecht University, Utrecht, Netherlands

**Keywords:** ESBL, pAmpC, *Escherichia coli*, plasmid, livestock

## Abstract

Extended-spectrum β-lactamase (ESBL) and plasmid-mediated AmpC β-lactamase (pAmpC) genes confer resistance to extended spectrum cephalosporin’s. The spread of these genes is mostly facilitated by plasmid-mediated horizontal transfer. National surveillance activities to detect ESBL/pAmpC-producers in commensal bacteria from livestock are in place in the Netherlands since several years. This study aimed at reporting gene and plasmid diversity of commensal ESBL/pAmpC-producing *Escherichia coli* isolated from healthy animals during surveillance activities between 2007 and 2017. A collection of 2304 extended-spectrum cephalosporin-resistant (ESC-R) *E. coli* isolated from feces of broilers, dairy cattle, slaughter pigs, turkeys, ducks, and veal calves was investigated and ESBL/pAmpC genes were determined. Gene location of a selection of 473 *E. coli* isolates was determined and typing of plasmids linked to the ESBL/pAmpC genes was performed. Twenty-two different ESBL/pAmpC genes were identified with *bla*_CTX-M-1_ being the most prevalent gene in livestock (43.7%), followed by *bla*_CMY -2_ and *bla*_SHV -12_, independent of the animal source. Prevalence of typically human associated *bla*_CTX-M-15_ was highest in cattle. Less than 10% *E. coli* isolates owed their ESC-R phenotype to promoter mutations of the chromosomal *ampC* gene. Majority (92%) of ESBL/pAmpC genes analyzed were plasmid located, with IncI1α being the most represented plasmid family in isolates from all animals, followed by IncF (veal calves, dairy cattle and slaughter pigs), IncK (broilers and laying hens), IncX1 in broilers, and emerging IncX3 in broilers and dairy cattle. Prevalence and molecular diversity of ESC-R *E. coli* isolated from livestock over an 11-year period revealed a composite scenario of gene-plasmid combinations.

## Introduction

Extended-spectrum β-lactamases (ESBLs) and plasmid-mediated AmpC β-lactamases (pAmpCs) are able to hydrolyse a large variety of β-lactam antibiotics, including cephalosporins and monobactams. The most clinically significant ESBL variants belong to the *bla*_CTX-M_, *bla*_TEM_, and *bla*_SHV_ gene families together with pAmpC *bla*_CMY_ gene family (Bush and Fisher, 2011). The successful spread of ESBL/pAmpC genes is mostly due to their localization on plasmids, resulting in easy transmission between bacteria (Rozwandowicz et al., 2018).

Extended-spectrum cephalosporin-resistant (ESC-R) Enterobacteriaceae have emerged globally in livestock animals during the last decades (Carattoli, 2008), with the consequent concern of animals being a putative source of ESBL/pAmpC-producing bacteria for humans either by direct contact or consumption of contaminated food products, as reviewed by (Ewers et al., 2012). Over the years, measures were implemented to reduce the use of third generation cephalosporins in livestock at national and European level (Speksnijder et al., 2015). Although the impact of transmission from livestock and the food chain on infections in humans is still debated (Madec et al., 2017; Dorado-Garcia et al., 2018), the ESBL/pAmpC reservoir in commensal bacteria from livestock has been increasingly investigated for its potential risk to public health (Michael et al., 2015).

Commensal ESC-R *Escherichia coli* randomly isolated from livestock feces have been monitored in the Netherlands since 1998, and phenotypic and genotypic results have annually been reported in the Monitoring of Antimicrobial Resistance and Antibiotic Usage in Animals in the Netherlands reports (MARAN Reports). Since 2014, active monitoring through selective culturing and reporting of antimicrobial resistance in several bacteria, including ESC-R *E. coli,* has become mandatory for member states of the European Union (European Food Safety Authority, 2008, 2013). Results of these activities are yearly published (Maran Reports, 2002/2017) but lack detailed information on plasmid typing and epidemiology.

The aim of this study is to report gene and plasmid diversity observed in ESC-R *E. coli* isolated from healthy livestock from 2007 to 2017 during surveillance activities in the Netherlands.

## Materials and Methods

### Surveillance Activities

All ESC-R *E. coli* isolates included in this retrospective study originated from fecal samples of livestock collected during different surveillance activities in the Netherlands. Because surveillance activities have changed over the years in terms of sampling and methodologies, full details can be found in the yearly reports (Maran Reports, 2002/2017). Main differences between monitoring activities are briefly described here. Non-selective culturing (2007–2017) was performed by isolation of one randomly selected *E. coli* colony from a directly inoculated MacConkey agar plate without supplemented antibiotics, each isolate representing one epidemiological unit as prescribed by EFSA guidelines (European Food Safety Authority, 2008). Selective culturing (2014–2017) was performed by overnight incubation of fecal samples in Buffered Peptone Water (BPW) followed by sub-culturing on MacConkey agar plate supplemented with 1 mg/L cefotaxime, according to EURL-AR protocols^1^. Sampling of ESC-R *E. coli* via selective isolation was performed on fecal samples from broilers, veal calves, slaughter pigs and dairy cows (European Food Safety Authority, 2013). Outside of mandatory surveillance activities, additional sampling was performed for turkeys in 2011 and 2012 (usually excluded because of low production), laying hens in 2014 and 2016 (typically screened only for *Salmonella*), and ducks in 2016 (not included in the legislation). Furthermore, ESC-R *E. coli* isolates obtained during monitoring activities from 2011 to 2013 by the Netherlands Food and Consumer Product Safety Authority (NVWA) with selective culturing (O/N enrichment in BPW followed by selective isolation on MacConkey agar plate with 1 mg/L cefotaxime) of fecal samples from broilers, dairy cattle, slaughter pigs and veal calves were included.

Overall, this retrospective study comprises 2304 ESC-R *E. coli* (Table 1): 330 ESC-R *E. coli* from non-selective surveillance (2007–2017), 1580 ESC-R *E. coli* from selective surveillance (2014–2017), and 394 ESC-R *E. coli* from NVWA selective surveillance activities (2011–2013).

**Table 1:** ESC-R *E. coli* isolates included in this study.

Animal and surveillance type	Year											Grand Total

	2007	2008	2009	2010	2011^#^	2012^#^	2013^#^	2014	2015	2016	2017	
**Broilers**												1047
*Non-selective*	8	63	53	51	23	25	13	11	10	3	5	265
*Selective*					29			269	235	151	98	782
**Dairy cattle**												172
*Non-selective*		2	2	1		1			1			7
*Selective*					14	7	3	26	33	46	36	165
**Ducks**												13
*Selective*										13		13
**Laying hens**												127
*Selective*								67		60		127
**Slaughter pigs**												439
*Non-selective*		3	11	1	3		4	2	1	1		26
*Selective*					64	67	46	72	56	61	47	413
**Turkeys**												19
*Non-selective*					18	1						19
**Veal calves**												487
*Non-selective*	2	2	2	1	2	1	1	1		1		13
*Selective*					68	60	36	54	43	99	114	474
**Grand Total**	**10**	**70**	**68**	**54**	**221**	**162**	**103**	**502**	**379**	**435**	**300**	**2304**


*^#^Monitoring activities by the NVWA*.

### Gene and Plasmid Typing

Along the years, different methods to identify ESBL/pAmpC gene families in ESC-R *E. coli* have been employed, including miniaturized DNA Microarrays (Identibac AMR-ve, Alere Technologies GmbH) (Batchelor et al., 2008), microarray analysis using the Check-MDR CT-101 array platform (Check-Points, Wageningen, Netherlands) or dedicated PCRs (Geurts et al., 2017). DNA was extracted by using the DNeasy Blood and Tissue kit (QIAGEN, Hilden, Germany) according to the manufacturer’s recommendations or DNA lysate preparation (Veldman et al., 2018). Independent of the screening method applied, gene sequences were confirmed by PCR amplification and DNA sequencing (Liakopoulos et al., 2016). Nucleotide and deduced amino acid sequences were compared with sequences in the Lahey clinic database^2^ and GenBank. Chromosomal mutations of promoters and attenuators of *ampC* genes were determined by sequencing and compared to GenBank (Mulvey et al., 2005).

A subset of 473 ESC-R *E. coli* was selected for genomic localization of ESBL/pAmpC genes: 63 from non-selective surveillance and 410 isolates from selective surveillance (Table 2). Over the years, different selection criteria were applied with the aim of including all ESBL/pAmpC genes detected in each animal species, and taking into consideration existing knowledge of gene-plasmid epidemiology. The chosen number of isolates per gene type was dependent on how prevalent the gene was in a given year i.e., for selective surveillance of broilers in 2014, 43% of samples were *bla*_CTXM-1_ positive (*n* = 116 out of 269), and a third of them (*n* = 42) were analyzed for genomic localization of *bla*_CTXM-1_ gene. For non-selective surveillance based on the knowledge that *bla*_CTXM-1_ positive *E. coli* in broilers are usually associated with IncI1 plasmids (Dierikx et al., 2013), majority of these isolates were not typed. Further details can be found in the corresponding results sections. ESC-R *E. coli* from 2011 to 2012 were not typed because of a temporary change in research priorities. Plasmid typing results for ESC-R *E. coli* isolated in 2017 via selective surveillance were not available at the time of writing.

**Table 2:** Selection of ESC-R *E. coli* examined to determine the genomic localization of ESBL/pAmpC genes.

Animal and surveillance type	Year								Grand Total

	2007	2008	2009	2010	2011	2014	2015	2016	
**Broilers**									197
*Non-selective*		9	9	15	5				38
*Selective*						99		60	159
**Dairy cattle**									29
*Non-selective*		1	2						3
*Selective*							26		26
**Ducks**									13
*Selective*								13	13
**Laying hens**									52
*Selective*						24		28	52
**Slaughter pigs**									79
*Non-selective*		3	11	1	2				17
*Selective*					25		37		62
**Veal calves**									103
*Non-selective*		1	2		2				5
*Selective*					58		40		98
**Grand Total**		**14**	**24**	**16**	**92**	**123**	**103**	**101**	**473**


Transformation experiments to assess plasmid location of ESBL/pAmpC genes and plasmid classification by PCR-based replicon typing (Carattoli et al., 2005) were performed according to standard procedures, as previously described (Liakopoulos et al., 2016). When transformants could not be retrieved, chromosomal location of ESBL/pAmpC genes was confirmed by I-CeuI Pulsed-Field Gel Electrophoresis (PFGE) of total bacterial DNA, followed by Southern blot hybridization, as previously described (Liu et al., 1993).

## Results and Discussion

### Overview of ESBL/pAmpC Gene Distribution Among Animal Species

**FIGURE 1:**
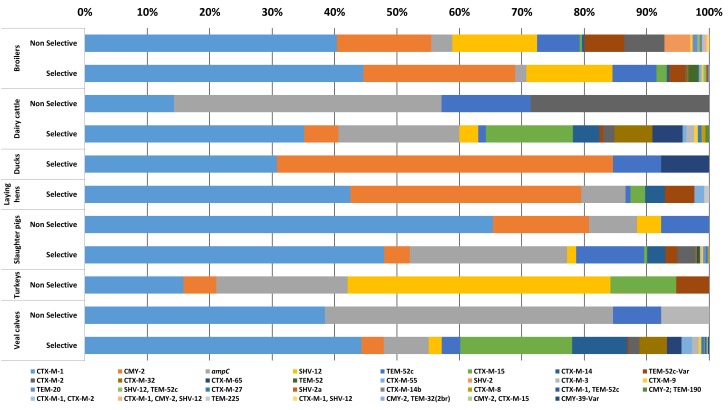
ESC-R *E. coli* gene distribution from selective and non-selective monitoring (2007–2017, *n* = 2304). Refer to Table 1 for isolate numbers for each livestock species and to Supplementary Table S1 for % values of each gene variant.

In 2087 out of 2304 *E. coli* isolates (90.5%), the ESC-R phenotype was associated with one or more ESBL/pAmpC genes (Figure 1). In total twenty-two different ESBL/pAmpC genes were detected. *bla*_CTX-M-1_ was the most prevalent (43.7%) independent of the animal origin, with the highest frequency observed in isolates from slaughter pigs (49%). Genes *bla*_CMY -2_ (14.4%), *bla*_TEM-52_ and variants (9.7%), *bla*_SHV -12_ (7.6%), and *bla*_CTX-M-15_ (5.6%) followed, the latter being the second most prevalent in veal calf (17.5%) after *bla*_CTX-M-1_ (44.1%). Eleven *E. coli* isolates showed co-presence of two ESBL/pAmpC genes: *bla*_CTX-M-1_ with *bla*_CTX-M-2_ (1 broiler), *bla*_SHV -12_ (1 broiler), or *bla*_TEM-52c_ (1 broiler and 1 veal calf); *bla*_SHV -12_ with *bla*_TEM-52c_ (4 broilers); and *bla*_CMY -2_ with *bla*_CTX-M-15_ (1 veal calf), *bla*_TEM-32_ (1 broiler) or *bla*_TEM-190_ (1 dairy cattle). One *E. coli* isolated from broiler encoded three genes: *bla*_CTX-M-1_, *bla*_CMY -2_, and *bla*_SHV -12._ In general, comparison of ESC-R *E. coli* derived from selective and non-selective surveillance of the same animal species showed a substantial difference in gene diversity, with selective culturing displaying higher gene variability (Figure 1). This difference is likely dependent on the significantly higher number of isolates available from selective surveillance than from the non-selective one (Table 1).

ESC-R *E. coli* for which no ESBL/pAmpC genes could be detected (*n* = 217, 9.4%) owed their ESC-R phenotype to promoter mutations of the chromosomally encoded *ampC* gene, with a peak observed in slaughter pigs (24.1%) (Figure 1). All *ampC* variants harbored previously described mutations (*ampC* types 2, 3, 5, 11, 18, 34, 40, and 45) (Mulvey et al., 2005). Majority of chromosomal *ampC* alterations (88%) created an alternate displaced promoter (type 3) whose mutation at position -42 is thought to have large effect on promoter strength (Caroff et al., 2000).

**FIGURE 2:**
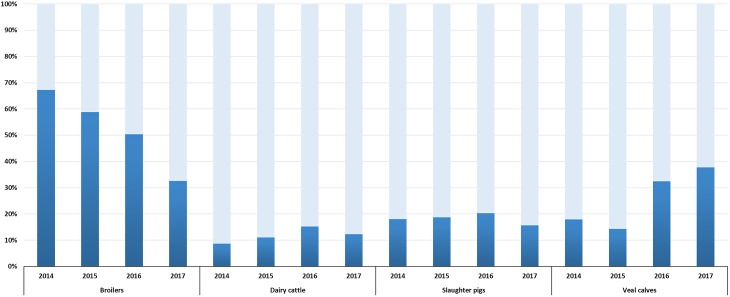
ESC-R *E. coli* prevalence in broilers, dairy cattle, slaughter pigs and veal calves from selective surveillance (2014–2017). Light gray bars indicate samples negative for the isolation of ESC-R *E. coli*. Refer to Supplementary Table S2 for isolate numbers and for each livestock species.

Selective surveillance from 2014 onward is based on the use of a harmonized protocol (European Food Safety Authority, 2008), a comparable number of isolates (300–400, depending on the year of sampling) and data are available for a four year period (2014–2017). Therefore, trends in ESC-R *E. coli* prevalence could be defined (Figure 2). A significant reduction (*p* < 0.001) from 67.3% (95% CI, 62.4–71.8) to 32.6% (95% CI, 27.3–38.2) was observed in broilers between 2014 and 2017. This trend is in accordance with decreasing prevalence of ESC-R *E. coli* from non-selective surveillance and in fresh chicken meat (Veldman et al., 2018). Prevalence in dairy cattle and slaughter pigs showed non-significant (*p* = 0.028, *p* = 0.2704) fluctuations between 2014 and 2017. A significant increase (*p* < 0.001) in prevalence was observed in veal calves, from 17.9% (95% CI, 13.8–22.8) in 2014 to 37.8% (95% CI, 32.3–43.5) in 2017. The prevalence of ESC-R in *E. coli* isolated from veal calves showed an increasing trend already in the years 1997–2010 (Hordijk et al., 2013b); this unexpected increase of ESC-R *E. coli* in veal calves urges for more research to define possible causes. Interestingly, beside the ubiquitous *bla*_CTX-M-1_, prevalence of *bla*_CTX-M-15_ and *bla*_CTX-M-14_ (ESBLs more associated with human *E. coli*) was dominant in cattle (veal calves and dairy cattle) (Supplementary Figure S1), a trend already observed since 2005 (Hordijk et al., 2013b) and confirmed in 2017 (Veldman et al., 2018). These findings are in line with recent studies showing that the general human population in the Netherlands has relatively similar ESBL gene profiles to veal calves compared to other reservoirs (Dorado-Garcia et al., 2018).

### Genomic Localization of ESBL/pAmpC Genes in ESC-R *E. coli* From Non-selective Surveillance

Genomic location (plasmid or chromosome) of a subset of ESBL/pAmpC genes was determined in 63 ESC-R *E. coli* collected over the years with non-selective culturing (Table 2). All ESBL/pAmpC genes were encoded on plasmids with different rep types (B/O, F, I1, K, and X1), in a few cases with multi-replicon plasmids (P/HI2 and P/I1), with distinctive prevalent gene-plasmid combinations per animal species (Supplementary Figure S2). Overall, the most common gene-plasmid combination was IncI1-*bla*_CTX-M-1_, detected in 30.1% of the isolates independently on the animal source. In broilers, IncX1-*bla*_TEM-52c-V ar_ and IncI1-*bla*_SHV -12_ were the most prevalent (36.8 and 39.5%, respectively) among the subset of analyzed isolates, excluding *bla*_CTX-M-1_ encoding *E. coli* that were not typed because typically associated with IncI1 plasmids (Dierikx et al., 2013). An *E. coli* isolate from broiler encoding genes *bla*_CTX-M-1_, *bla*_CMY -2_, and *bla*_SHV -12_ was associated to three IncI1, IncK, and IncX3 plasmids, respectively (Veldman et al., 2012). ESC-R Enterobacteriaceae encoding multiple ESBL/pAmpC genes have been described previously with various genomic settings both on plasmids and/or chromosome in livestock, meat, and clinical isolates (Dhanji et al., 2010; Veldman et al., 2010; Huang et al., 2017), depicting the complex plasmid scenario of cephalosporin-resistance circulation among Enterobacteriaceae. Vast majority of ESC-R *E. coli* isolates from slaughter pigs were associated to IncI1 plasmids carrying *bla*_CTX-M-1_ (76.5%). The most common gene-plasmid combination in veal calves isolates was IncI1-*bla*_CTX-M-1_, while ESC-R *E. coli* isolates from dairy cattle were associated with multi-replicon plasmids IncP/HI2 encoding *bla*_CTX-M-2_. All IncI1 plasmid subtyped (89%) were confirmed to be IncI1α (data not shown).

### Genomic Localization of ESBL/pAmpC Genes in ESC-R *E. coli* From Selective Surveillance

According to current guidelines (European Food Safety Authority, 2013), selective surveillance of ESC-R *E. coli* should be performed following an annual rotation system: broilers and turkeys (years 2014, 2016, 2018, 2020), pigs and bovines (years 2015, 2017, 2019). Although more animal species than the recommended ones are frequently analyzed in the Dutch surveillance program (Maran Reports, 2002/2017), the rotation system was followed to select a subset of ESC-R *E. coli* (*n* = 410) for further investigation on the genomic localization of ESBL/pAmpC genes (Table 2). Because poultry ESC-R isolates for 2014 and 2016 were too many to include in the analysis (*n* = 783), 40–50% of all *E. coli* from broilers (*n* = 99 and *n* = 60, respectively) and laying hens (*n* = 24 and *n* = 28, respectively) were screened per year.

**FIGURE 3:**
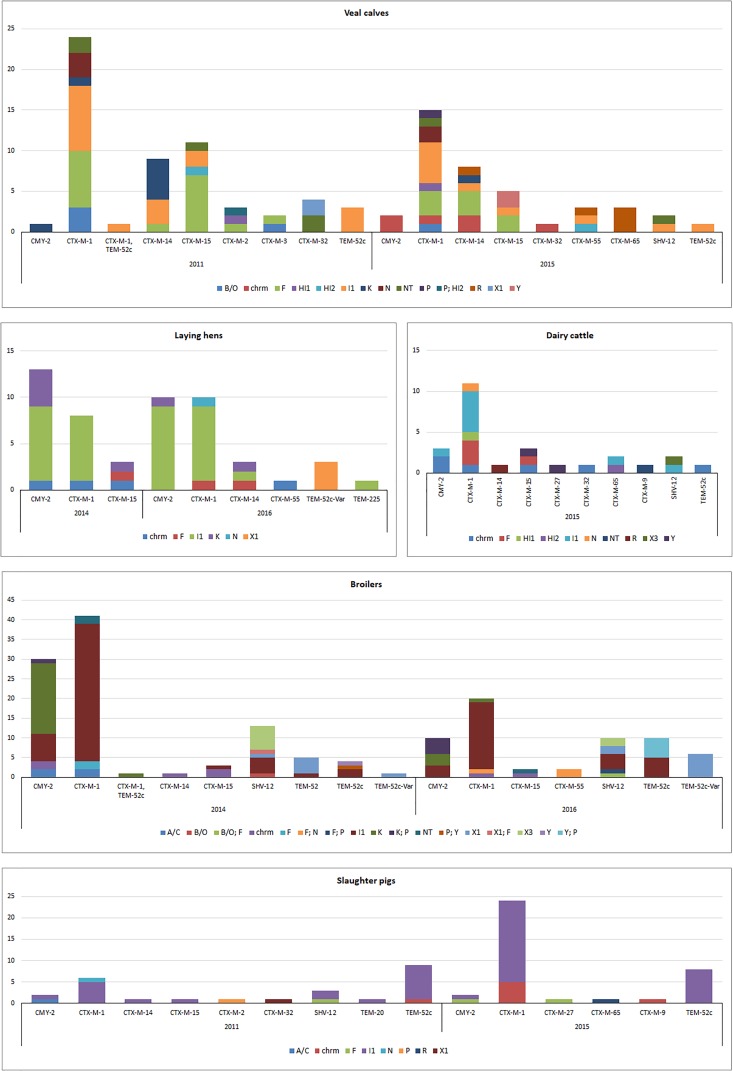
Genomic localization of ESBL/pAmpC genes in ESC-R *E. coli* from selective monitoring (2011–2016) per animal species. Panels: veal calves (*n* = 98); broilers (*n* = 159); laying hens (*n* = 52); dairy cattle (*n* = 26); slaughter pigs (*n* = 62). Refer to Supplementary Table S3 for % values of each gene variant in different livestock species.

Results of this analysis are reported in Figure 3, except for thirteen ESC-R *E. coli* from ducks (2016) characterized by chromosomal *bla*_CMY -2_ (*n* = 8) or IncI1 plasmids carrying *bla*_CTX-M-1_ (*n* = 5). Transformants for 38 (9.3%) ESC-R *E. coli* could not be recovered. PFGE and Southern hybridization confirmed the chromosomal location of ESBL/pAmpC genes, mostly belonging to the CTX-M group (Figure 3): *bla*_CTX-M-1_, *bla*_CTX-M-14_, *bla*_CTX-M-15_, *bla*_CTX-M-32_, *bla*_CTX-M-55_, *bla*_CTX-M-9_, and *bla*_CMY -2_. Although, the genetic surroundings of these genes were not investigated, it is known that IS*Ecp*1 insertion sequence upstream of ESBL/pAmpC genes are associated with transposition and chromosomal integration of typically plasmid-encoded genes in *E. coli*, *K. pneumoniae*, and *Shigella* *flexneri*, among others, from animals or humans (Wang et al., 2013; Fang et al., 2015; Huang et al., 2017). Through chromosomal integration, IS*Ecp*1 might contribute to lowering the fitness cost derived from harboring an entire plasmid, while enhancing EBSL/AmpC gene expression under its own promoter (Poirel et al., 2003).

Majority of ESBL/pAmpC genes (*n* = 372) were associated with plasmids (Figure 3). All IncI1 plasmid subtyped (86%) were confirmed to be IncI1α (data not shown). Gene-plasmid combinations in broilers did not show major differences between 2014 and 2016. IncI1-*bla*_CTX-M-1_ plasmids were the most common, followed by IncI1 encoding *bla*_CMY -2_, *bla*_SHV -12_ or *bla*_TEM-52c_. IncK-*bla*_CMY -2_ (or multireplicon IncK/P) plasmids were also commonly detected, suggesting a relatively stable plasmid population in broilers in the Netherlands, as earlier described (Dierikx et al., 2013). Yet, IncX3-*bla*_SHV -12_ plasmids, whose emergence in Dutch ESC-R *E. coli* of animal origin was recently revealed alongside a gradual decrease in the prevalence of IncI1-*bla*_SHV -12_ plasmids (Liakopoulos et al., 2018), were detected in both years. Plasmid IncX1 carrying *bla*_SHV -12,_ *bla*_TEM-52c_ or *bla*_TEM-52c-V ar_ followed in prevalence, the latter detected also in ESBL/pAmpC-producing *E. coli* from laying hens in 2016. Overall, plasmid-gene associations in isolates from laying hens were comparable to broilers, with IncI1-*bla*_CMY -2,_ IncI1-*bla*_CTX-M-1_ and IncK-*bla*_CMY -2_ being the most predominant in both 2014 and 2016. The presence of ESC-R *E. coli* at all levels of the Dutch broiler production pyramid has been demonstrated, as day-old chicks can inherit bacteria from their parents through contaminated egg shells or from the environment (Dierikx et al., 2013).

ESBL/pAmpC-producing *E. coli* isolated from slaughter pigs from both 2011 and 2015 were dominated by IncI1 plasmids encoding *bla*_CTX-M-1_ or *bla*_TEM-52c_, recognized as the most prevalent gene-plasmid combinations in Enterobacteriaceae from slaughter pigs worldwide (Geser et al., 2011; Randall et al., 2014; Biasino et al., 2018; Dang et al., 2018). ESC-R *E. coli* isolates from dairy cattle and veal calves showed a quite variable array of plasmid-gene combinations (Figure 3). Beside predominant IncI1 plasmids, IncF plasmids were detected in both animal reservoirs in association with *bla*_CTX-M-1_ and *bla*_CTX-M-14_ and *bla*_CTX-M-15_ genes. IncR-*bla*_CTX-M-65_ and IncR-*bla*_CTX-M-55_ were also identified in veal calves in 2015 but no R plasmid was detected in 2011. Gene-plasmid combinations observed in veal calves are coherent with previous studies conducted in the Netherlands and in France (Hordijk et al., 2013a; Haenni et al., 2014) with relatively high prevalence of various *bla*_CTX-M_ genes located on IncF and IncI1 plasmids. The more variable array of plasmid-gene combinations observed in veal calves compared to other livestock might be a consequence of international trade from different dairy farms to Dutch farms as well as high antimicrobial use and farm management.

In conclusion, the results of this study provide insight in the prevalence and molecular diversity of ESC-R *E. coli*, revealing a rather composite scenario of plasmid-gene combinations circulating in livestock from the Netherlands over the last decade. Yet, the bias in the selection of isolates for plasmid typing should be kept in mind to avoid risky conclusions on prevalence of ESBL-harboring plasmid types. Nevertheless, the study provides additional information on the occurrence of different plasmid types carrying ESBL/pAmpC-genes in *E. coli* from livestock in the Netherlands. These findings also demonstrate the added value of selective culturing of ESC-R *E. coli* and genotyping of genes and plasmids over random isolation for resistance determinants of public health concern.

## Author Contributions

AK, AE-Z, JH, and BW acquired the data. CD, DC, KV and DM analyzed the data. DC and KV prepared the manuscript. All authors discussed, read, contributed to, and approved the final manuscript.

## Conflict of Interest Statement

The authors declare that the research was conducted in the absence of any commercial or financial relationships that could be construed as a potential conflict of interest.

## Footnotes

1 http://www.eurl-ar.eu/233-protocols.htm

2 http://www.lahey.org/Studies

## Supplementary Material

FIGURE S1 Gene distribution in ESC-R *E. coli* isolated from broilers, dairy cattle, slaughter pigs and veal calves during selective surveillance (2014–2017). Refer to Supplementary Table S2 for gene distribution for each livestock species.

FIGURE S2 Gene-plasmid combinations in ESC-R *E. coli* from non-selective surveillance per animal species (2008–2011, n = 63). Refer to Supplementary Table S4 for gene-plasmid distribution for each livestock species.

TABLE S1 % values of ampC and ESBL/pAmpC genes distribution from selective and non-selective monitoring (2007–2017, n = 2304).

TABLE S2 ESC-R E. coli prevalence in broilers, dairy cattle, slaughter pigs and veal calves from selective surveillance (2014–2017).

TABLE S3 Genomic localization of ESBL/pAmpC genes in ESC-R E. coli from selective surveillance per animal species (2011–2016, n = 410).

TABLE S4 Gene-plasmid combinations in ESC-R E. coli from non-selective surveillance per animal species (2008–2011, n = 63).
