# Supplementary figures and images for: Diversity of Plasmids and Genes Encoding Resistance to Extended Spectrum Cephalosporins in Commensal Escherichia coli From Dutch Livestock in 2007–2017
Source: Front Microbiol. 2019 Feb 4;10:76. doi: 10.3389/fmicb.2019.00076 (PMC6369715; doi:10.3389/fmicb.2019.00076)

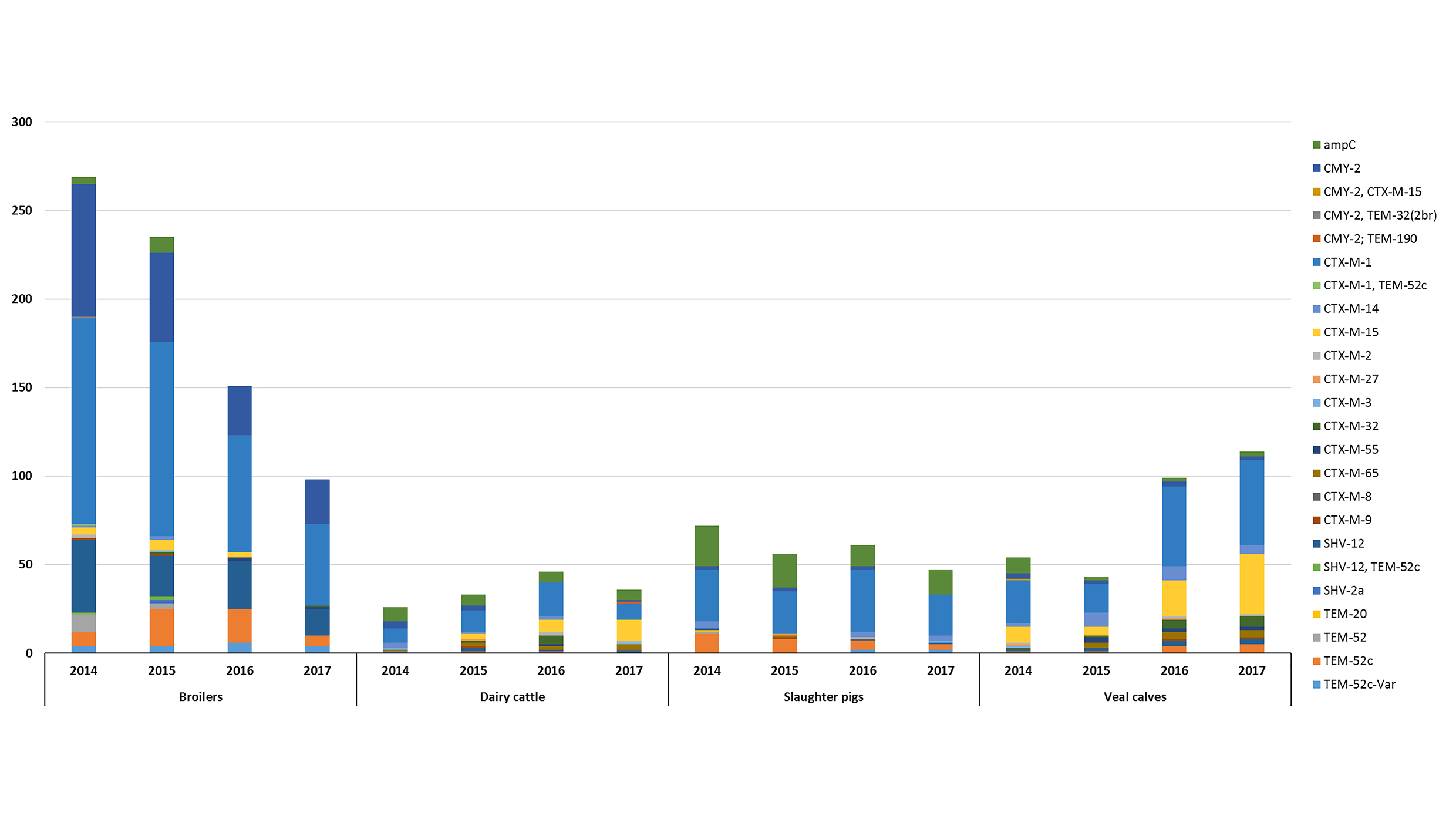

Supplement: FIGURE S1 — Gene distribution in ESC-R E. coli isolated from broilers, dairy cattle, slaughter pigs and veal calves during selective surveillance (2014–2017). Refer to Supplementary Table S2 for gene distribution for each livestock species. [file Image_1.TIF]

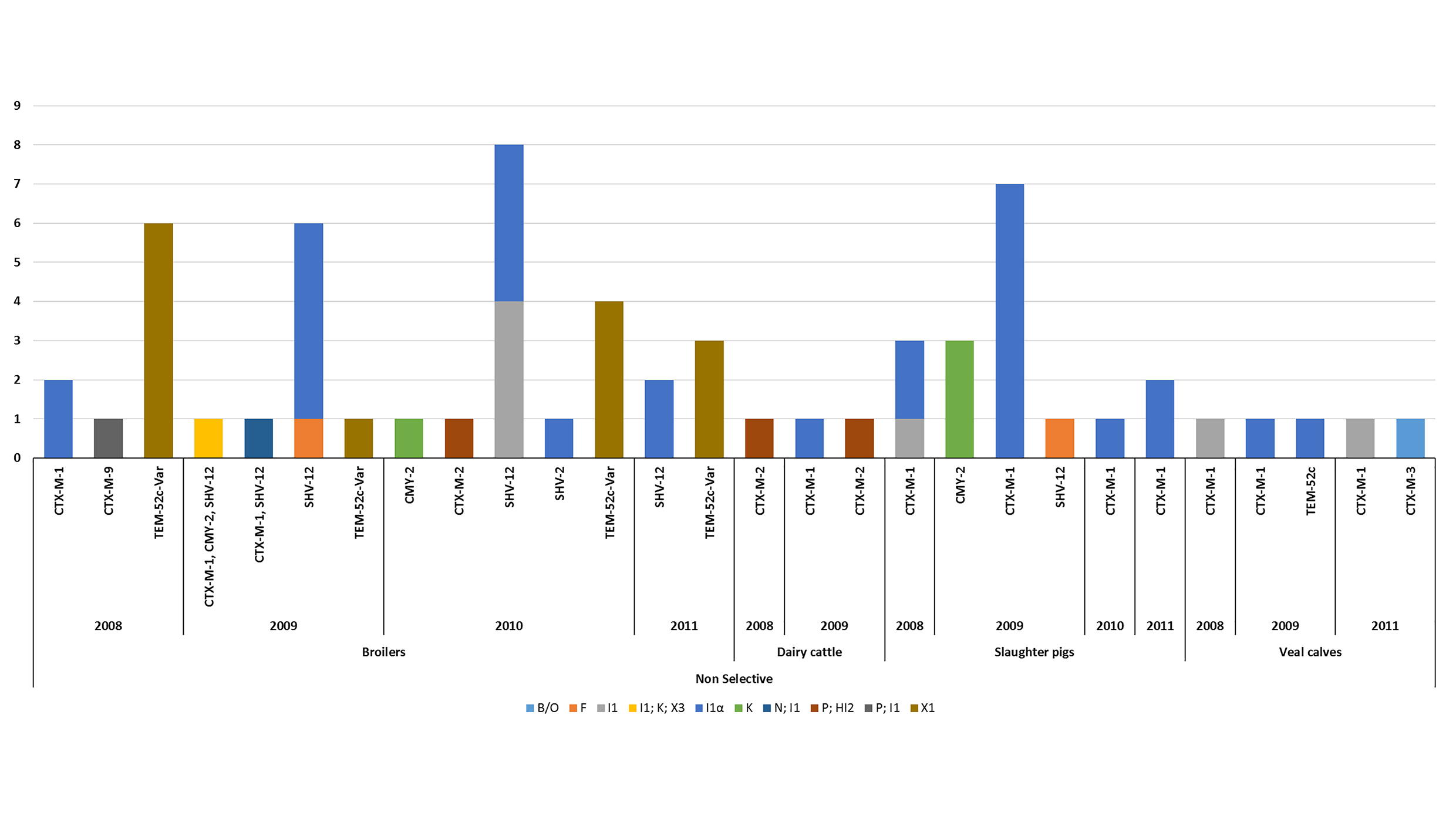

Supplement: FIGURE S2 — Gene-plasmid combinations in ESC-R E. coli from non-selective surveillance per animal species (2008–2011, n = 63). Refer to Supplementary Table S4 for gene-plasmid distribution for each livestock species. [file Image_2.TIF]
