# Supplementary material for: Diversity of Plasmids and Genes Encoding Resistance to Extended Spectrum Cephalosporins in Commensal Escherichia coli From Dutch Livestock in 2007–2017
Source: Front Microbiol. 2019 Feb 4;10:76. doi: 10.3389/fmicb.2019.00076 (PMC6369715; doi:10.3389/fmicb.2019.00076)
Supplement: TABLE S1 — % values of ampC and ESBL/pAmpC genes distribution from selective and non-selective monitoring (2007–2017, n = 2304). [file Data_Sheet_1.PDF]

Table S1. % values of *ampC* and ESBL/pAmpC genes distribution from selective and non-selective monitoring (2007-2017, n=2304).
Supporting information to Figure 1.

Livestock (method)	CTX-M-1	CMY-2	*ampC*	SHV-12	TEM-52c	CTX-M-15	CTX-M-14	TEM-52c-Var	CTX-M-2	CTX-M-32	CTX-M-65	TEM-52	CTX-M-55	SHV-2	CTX-M-3	CTX-M-9	TEM-20	SHV-12, TEM-52c	CTX-M-27	SHV-2a	CTX-M-14b	CTX-M-8	CTX-M-1, TEM-52c	CMY-2; TEM-190	CTX-M-1, CTX-M-2	CTX-M-1, CMY-2, SHV-12	TEM-225	CTX-M-1, SHV-12	CMY-2, TEM-32(2br)	CMY-2, CTX-M-15	CMY-39-Var
**Broilers**																															
Non Selective	40.4%	15.1%	3.4%	13.6%	6.8%	0.4%	0.4%	6.4%	6.4%	0.0%	0.0%	0.0%	0.0%	4.2%	0.0%	0.4%	0.8%	0.4%	0.0%	0.0%	0.4%	0.0%	0.0%	0.0%	0.4%	0.4%	0.0%	0.4%	0.0%	0.0%	0.0%
Selective	44.6%	24.3%	1.8%	13.8%	7.0%	1.7%	0.4%	2.6%	0.3%	0.3%	0.0%	1.7%	0.4%	0.0%	0.1%	0.3%	0.0%	0.4%	0.0%	0.3%	0.0%	0.0%	0.1%	0.0%	0.0%	0.0%	0.0%	0.0%	0.1%	0.0%	0.0%
**Dairy cattle**																															
Non Selective	14.3%	0.0%	42.9%	0.0%	14.3%	0.0%	0.0%	0.0%	28.6%	0.0%	0.0%	0.0%	0.0%	0.0%	0.0%	0.0%	0.0%	0.0%	0.0%	0.0%	0.0%	0.0%	0.0%	0.0%	0.0%	0.0%	0.0%	0.0%	0.0%	0.0%	0.0%
Selective	35.2%	5.5%	19.4%	3.0%	1.2%	13.9%	4.2%	0.6%	1.8%	6.1%	4.8%	0.0%	0.6%	0.0%	1.2%	0.6%	0.0%	0.0%	0.6%	0.0%	0.0%	0.6%	0.0%	0.6%	0.0%	0.0%	0.0%	0.0%	0.0%	0.0%	0.0%
**Ducks**																															
Selective	30.8%	53.8%	0.0%	0.0%	7.7%	0.0%	0.0%	0.0%	0.0%	0.0%	7.7%	0.0%	0.0%	0.0%	0.0%	0.0%	0.0%	0.0%	0.0%	0.0%	0.0%	0.0%	0.0%	0.0%	0.0%	0.0%	0.0%	0.0%	0.0%	0.0%	0.0%
**Laying hens**																															
Selective	42.5%	37.0%	7.1%	0.0%	0.8%	2.4%	3.1%	4.7%	0.0%	0.0%	0.0%	0.0%	1.6%	0.0%	0.0%	0.0%	0.0%	0.0%	0.0%	0.0%	0.0%	0.0%	0.0%	0.0%	0.0%	0.0%	0.8%	0.0%	0.0%	0.0%	0.0%
**Slaughter pigs**																															
Non Selective	65.4%	15.4%	7.7%	3.8%	7.7%	0.0%	0.0%	0.0%	0.0%	0.0%	0.0%	0.0%	0.0%	0.0%	0.0%	0.0%	0.0%	0.0%	0.0%	0.0%	0.0%	0.0%	0.0%	0.0%	0.0%	0.0%	0.0%	0.0%	0.0%	0.0%	0.0%
Selective	47.9%	4.1%	25.2%	1.5%	10.9%	0.5%	2.9%	1.9%	2.9%	0.2%	0.2%	0.2%	0.0%	0.0%	0.2%	0.2%	0.5%	0.0%	0.2%	0.0%	0.0%	0.2%	0.0%	0.0%	0.0%	0.0%	0.0%	0.0%	0.0%	0.0%	0.0%
**Turkeys**																															
Non Selective	15.8%	5.3%	21.1%	42.1%	0.0%	10.5%	0.0%	5.3%	0.0%	0.0%	0.0%	0.0%	0.0%	0.0%	0.0%	0.0%	0.0%	0.0%	0.0%	0.0%	0.0%	0.0%	0.0%	0.0%	0.0%	0.0%	0.0%	0.0%	0.0%	0.0%	0.0%
**Veal calves**																															
Non Selective	38.5%	0.0%	46.2%	0.0%	7.7%	0.0%	0.0%	0.0%	0.0%	0.0%	0.0%	0.0%	0.0%	0.0%	7.7%	0.0%	0.0%	0.0%	0.0%	0.0%	0.0%	0.0%	0.0%	0.0%	0.0%	0.0%	0.0%	0.0%	0.0%	0.0%	0.0%
Selective	44.3%	3.6%	7.2%	2.1%	3.0%	17.9%	8.9%	0.2%	1.7%	4.4%	2.3%	0.0%	1.7%	0.0%	1.1%	0.4%	0.0%	0.0%	0.4%	0.0%	0.2%	0.0%	0.2%	0.0%	0.0%	0.0%	0.0%	0.0%	0.0%	0.2%	0.2%

Table S2. ESC-R *E. coli* prevalence in broilers, dairy cattle, slaughter pigs and veal calves from selective surveillance (2014-2017).
Supporting information to Figure 2 and Figure S1.

Livestock	Year	Total samples	TEM-52c-Var	TEM-52c	TEM-52	TEM-20	SHV-2a	SHV-12, TEM-52c	SHV-12	CTX-M-9	CTX-M-8	CTX-M-65	CTX-M-55	CTX-M-32	CTX-M-3	CTX-M-27	CTX-M-2	CTX-M-15	CTX-M-14	CTX-M-1, TEM-52c	CTX-M-1	CMY-2; TEM-190	CMY-2, TEM-32(2br)	CMY-2, CTX-M-15	CMY-2	Chrm ampC	Grand Total ESCR (%)
**Broilers**	**2014**	400	4	8	10			1	41	1							2	4	1	1	116		1		75	4	269 (67.3)
	**2015**	400	4	21	3		2	2	23	1				1	1			6	2		110				50	9	235 (58.8)
	**2016**	300	6	19					27				2					3			66				28		151 (50.3)
	**2017**	301	4	6					15				1	1							46				25		98 (32.6)
**Dairy cattle**	**2014**	300	1									1			1				3		8				4	8	26 (8.7)
	**2015**	300		1					2	1		2		1		1		3	1		12				3	6	33 (11)
	**2016**	302		1					1			2	1	5			2	7	2		19					6	46 (15.2)
	**2017**	292							1		1	3			1		1	12			9	1			1	6	36 (12.3)
**Slaughter pigs**	**2014**	400		11	1	1			1										4		29				2	23	72 (18)
	**2015**	300		8						1		1				1					24				2	19	56 (18.7)
	**2016**	300	2	5							1						1		3		35				2	12	61 (20.3)
	**2017**	300	2	3					1						1				3		23					14	47 (15.7)
**Veal calves**	**2014**	301		1					1					1	1	1	1	9	2		24			1	3	9	54 (17.9)
	**2015**	300		1					2			3	3	1				5	8		16				2	2	43 (14.3)
	**2016**	305		4					3	1		4	2	5		1	1	20	8		45				3	2	99 (32.5)
	**2017**	302		5					3	1		4	2	6			1	34	5		48				2	3	114 (37.7)

Table S3. Genomic localization of ESBL/pAmpC genes in ESC-R E. coli from selective surveillance per animal species (2011-2016, n=410). % are expressed in relation to each livestock/year
Supporting information to Figure 3.

Livestock/Year/Gene	A/C	B/O	B/O; F	chrm	F	F; N	F; P	HI1	HI2	I1	I1α	K	K; P	N	NT	P	P; HI2	P; Y	R	X1	X1; F	X3	Y	Y; P	Grand Total
**Broilers**	4	1	1	7	2	3	1			19	60	23	5		3			1		14	1	8	1	5	159
**2014**	4	1		5	2					19	31	19	1		2			1		6	1	6	1		99
CMY-2	2 (2.0 %)			2 (2.0 %)						4 (4.0%)	3 (3.0%)	18 (18.0%)	1 (1.0%)												30
CTX-M-1	2 (2.0 %)				2 (2.0 %)					13 (13.0%)	22 (22.0%)				2 (2.0 %)										41
CTX-M-1, TEM-52c												1 (1.0%)													1
CTX-M-14				1 (1.0%)																					1
CTX-M-15				2 (2.0 %)						1 (1.0%)															3
SHV-12		1 (1.0%)								1 (1.0%)	3 (3.0%)									1 (1.0%)	1 (1.0%)	6 (6.0%)			13
TEM-52											1 (1.0%)									4 (4.0%)					5
TEM-52c											2 (2.0 %)							1 (1.0%)					1 (1.0%)		4
TEM-52c-Var																				1 (1.0%)					1
**2016**			1	2		3	1				29	4	4		1					8		2		5	60
CMY-2											3 (5.0%)	3 (5.0%)	4 (6.7%)												10
CTX-M-1				1 (1.7%)		1 (1.7%)					17 (28.3%)	1 (1.7%)													20
CTX-M-15				1 (1.7%)											1 (1.7%)										2
CTX-M-55						2 (3.3%)																			2
SHV-12			1 (1.7%)				1 (1.7%)				4 (6.7%)									2 (3.3%)		2 (3.3%)			10
TEM-52c											5 (8.3%)													5 (8.3%)	10
TEM-52c-Var																				6 (10.0%)					6
**Dairy cattle**				6	4			1	1		8			1	1				1			1	2		26
**2015**				6	4			1	1		8			1	1				1			1	2		26
CMY-2				2 (7.7%)							1 (3.8%)														3
CTX-M-1				1 (3.8%)	3 (11.5%)			1 (3.8%)			5 (19.2%)			1 (3.8%)											11
CTX-M-14																			1 (3.8%)						1
CTX-M-15				1 (3.8%)	1 (3.8%)																		1 (3.8%)		3
CTX-M-27																							1 (3.8%)		1
CTX-M-32				1 (3.8%)																					1
CTX-M-65									1 (3.8%)		1 (3.8%)														2
CTX-M-9															1 (3.8%)										1
SHV-12											1 (3.8%)											1 (3.8%)			2
TEM-52c				1 (3.8%)																					1
**Ducks**				8							5														13
**2016**				8							5														13
CMY-2				6 (46.1%)							1 (7.7%)														7
CTX-M-1				2 (15.4%)							2 (15.4%)														4
CTX-M-65											1 (7.7%)														1
TEM-52c											1 (7.7%)														1
**Laying hens**				4	3					6	28	7		1						3					52
**2014**				3	1					6	9	5													24
CMY-2				1 (4.2%)						4 (16.7%)	4 (16.7%)	4 (16.7%)													13
CTX-M-1				1 (4.2%)						2 (8.3%)	5 (20.8%)														8
CTX-M-15				1 (4.2%)	1 (4.2%)							1 (4.2%)													3
**2016**				1	2						19	2		1						3					28
CMY-2											9 (32.1%)	1 (3.6)													10
CTX-M-1					1 (3.6)						8 (28.6%)			1 (3.6)											10
CTX-M-14					1 (3.6)						1 (3.6)	1 (3.6)													3
CTX-M-55				1 (3.6)																					1
TEM-225											1 (3.6)														1
TEM-52c-Var																				3 (10.7%)					3
**Slaughter pigs**	1			7	3					1	46			1		1			1	1					62
**2011**	1			1	1					1	18			1		1				1					25
CMY-2	1 (4.0%)										1 (4.0%)														2
CTX-M-1											5 (17.9%)			1 (4.0%)											6
CTX-M-14											1 (4.0%)														1
CTX-M-15											1 (4.0%)														1
CTX-M-2																1 (4.0%)									1
CTX-M-32																				1 (4.0%)					1
SHV-12					1 (4.0%)						2 (8.0%)														3
TEM-20											1 (4.0%)														1
TEM-52c				1 (4.0%)						1 (4.0%)	7 (28.0%)														9
**2015**				6	2						28								1						37
CMY-2					1 (2.7%)						1 (2.7%)														2
CTX-M-1				5 (13.5%)							19 (51.3%)														24
CTX-M-27					1 (2.7%)																				1
CTX-M-65																			1 (2.7%)						1
CTX-M-9				1 (2.7%)																					1
TEM-52c											8 (21.6%)														8
**Veal calves**		5		6	25			2	2	3	24	8		5	7	1	1		5	2			2		98
**2011**		4			17			1	1	3	14	7		3	5		1			2					58
CMY-2												1 (1.7%)													1
CTX-M-1		3 (5.2%)			7 (12.1%)					1 (1.7%)	7 (12.1%)	1 (1.7%)		3 (5.2%)	2 (3.4%)										24
CTX-M-1, TEM-52c											1 (1.7%)														1
CTX-M-14					1 (1.7%)						3 (5.2%)	5 (8.6%)													9
CTX-M-15					7 (12.1%)				1 (1.7%)	2 (3.4%)					1 (1.7%)										11
CTX-M-2					1 (1.7%)			1 (1.7%)									1 (1.7%)								3
CTX-M-3		1 (1.7%)			1 (1.7%)																				2
CTX-M-32															2 (3.4%)					2 (3.4%)					4
TEM-52c											3 (5.2%)														3
**2015**		1		6	8			1	1		10	1		2	2	1			5				2		40
CMY-2				2 (5.0%)																					2
CTX-M-1		1 (2.5%)		1 (2.5%)	3 (7.5%)			1 (2.5%)			5 (12.5%)			2 (5.0%)	1 (2.5%)	1 (2.5%)									15
CTX-M-14				2 (5.0%)	3 (7.5%)						1 (2.5%)	1 (2.5%)							1 (2.5%)						8
CTX-M-15					2 (5.0%)						1 (2.5%)												2 (5.0%)		5
CTX-M-32				1 (2.5%)																					1
CTX-M-55									1 (2.5%)		1 (2.5%)								1 (2.5%)						3
CTX-M-65																			3 (7.5%)						3
SHV-12											1 (2.5%)				1 (2.5%)										2
TEM-52c											1 (2.5%)														1
**Grand Total**	5	6	1	38	37	3	1	3	3	29	171	38	5	8	11	2	1	1	7	20	1	9	5	5	410

Table S4. Gene-plasmid combinations in ESC-R *E. coli* from non-selective surveillance per animal species (2008-2011, n=63). Supporting information to Figure S2.

Livestock/Year/Gene	B/O	F	I1	I1; K; X3	I1α	K	N; I1	P; HI2	P; I1	X1
**Broilers**										
**2008**										
CTX-M-1					100%					
CTX-M-9									100%	
TEM-52c-Var										100%
**2009**										
CTX-M-1, CMY-2, SHV-12				100%						
CTX-M-1, SHV-12							100%			
SHV-12		17%			83%					
TEM-52c-Var										100%
**2010**										
CMY-2						100%				
CTX-M-2								100%		
SHV-12			50%		50%					
SHV-2					100%					
TEM-52c-Var										100%
**2011**										
SHV-12					100%					
TEM-52c-Var										100%
**Dairy cattle**										
**2008**										
CTX-M-2								100%		
**2009**										
CTX-M-1					100%					
CTX-M-2								100%		
**Slaughter pigs**										
**2008**										
CTX-M-1			33%		67%					
**2009**										
CMY-2						100%				
CTX-M-1					100%					
SHV-12		100%								
**2010**										
CTX-M-1					100%					
**2011**										
CTX-M-1					100%					
**Veal calves**										
**2008**										
CTX-M-1			100%							
**2009**										
CTX-M-1					100%					
TEM-52c					100%					
**2011**										
CTX-M-1			100%							
CTX-M-3	100%									
